# Ridge-Furrow Planting and Water-Retaining Agents Improve Quinoa Yield via Enhanced Photosynthesis and Translocation

**DOI:** 10.3390/plants15131956

**Published:** 2026-06-25

**Authors:** Hongjie Zhang, Jinhu Yang, Jianqing Feng, Siyao Wang, Jiawei Wang, Ying Wang, Xinyao Zhao, Li Han, Yanli Zhang, Li Zhang, Dongjuan Wang, Lele Tian, Xiaorong Wu, Lijun Li

**Affiliations:** 1 College of Agronomy, Inner Mongolia Agricultural University, Huhhot 010019, China; zzhj@emails.imau.edu.cn (H.Z.); yjh0019@emails.imau.edu.cn (J.Y.); 15248328789@emails.imau.edu.cn (J.F.); 2024212020098@emails.imau.edu.cn (S.W.); 2024202020026@emails.imau.edu.cn (J.W.); nxywy123@imau.edu.cn (Y.W.); zxy0520@emails.imau.edu.cn (X.Z.); hanli@emails.imau.edu.cn (L.H.); tianlele@emails.imau.edu.cn (L.T.); 2 Institute of Resources, Environment and Sustainable Development, Inner Mongolia Academy of Agricultural & Animal Husbandry Sciences, Hohhot 010031, China; zyanli2010@126.com; 3 Animal Husbandry and Agricultural Technology Extension Center, Ongniud Banner, Chifeng 024500, China; 200513114@163.com (L.Z.); 15949432014@163.com (D.W.)

**Keywords:** *Chenopodium quinoa* Willd., ridge-furrow planting, superabsorbent polymer (SAP), source–sink relationship, photosynthetic capacity, dry-land agriculture, crop yield

## Abstract

Sustainable quinoa production in semi-arid regions is constrained by water scarcity and inefficient photoassimilate partitioning. This study evaluated the interactive effects of planting patterns and superabsorbent polymer (SAP) application rates (0, 60, and 90 kg·ha^−1^) on ‘Longli 5’ quinoa source–sink dynamics. Results demonstrated that ridge-furrow planting coupled with a moderate SAP rate (60 kg·ha^−1^, D1) synergistically optimized the soil moisture environment, enhancing the two-year average leaf area index (LAI) by 52.6–66.9% and net photosynthetic rate (Pn) by 5.45–10.64% (*p* < 0.05). Unlike the high SAP rate (90 kg·ha^−1^), “water luxury consumption” and low input efficiency dynamic which induced “water luxury consumption” and low input efficiency, the D1 treatment effectively promoted photoassimilate remobilization to grains by 13.39–14.44%. Unlike high SAP rates (90 kg·ha^−1^), which induced ‘water luxury consumption’ and low input efficiency, the D1 treatment effectively promoted photoassimilate remobilization to grains by 13.39–14.44%.This mechanism optimized source–sink coordination, resulting in a significant increase in seed yield and thousand-grain weight (*p* < 0.05). These findings conclude that a balanced source–sink allocation, rather than maximal source capacity alone, is the definitive driver of productivity. Integrating ridge-furrow planting with moderate SAP application (60 kg·ha^−1^) is a highly efficient agronomic strategy for harmonizing quinoa growth and achieving sustainable, high yields in semi-arid environments.

## 1. Introduction

Driven by a global population projected to reach nearly 10 billion by 2050, global food security faces unprecedented challenges that necessitate an estimated 60% increase in agricultural output [1,2,3]. Concurrently, climate change and recurrent extreme weather events have exacerbated water scarcity, rendering it the most critical abiotic factor limiting crop yields in semi-arid regions [4,5,6]. Because water deficits severely disrupt fundamental physiological functions—including photosynthesis, nutrient assimilation, and biomass allocation they pose a profound threat to agricultural stability [7,8,9,10,11,12]. Therefore, the integration of climate-resilient crops with innovative, sustainable agronomic practices is imperative to ensure stable food production under increasingly limited water resources.

Quinoa (*Chenopodium quinoa* Willd.), a pseudo-cereal native to the Andean region [13,14], has garnered global prominence as a “super crop” due to its exceptional nutritional profile comprising balanced essential amino acids and high-quality proteins—and its remarkable resilience to drought and salinity. However, despite its inherent physiological plasticity, the cultivation of quinoa in semi-arid regions remains constrained by erratic rainfall and severe water scarcity, often resulting in inconsistent productivity [15,16]. Traditional extensive farming practices are frequently inadequate to sustain optimal water use efficiency (WUE) [10,17]. Therefore, optimizing the field micro-environment through advanced water-saving techniques is essential to bridge the gap between quinoa’s genetic potential and its actual field performance in these fragile ecosystems.

Strategic modifications of field microtopography and the application of soil amendments represent robust approaches for alleviating drought-induced constraints on crop productivity [18]. Ridge-furrow planting, an established micro-rain harvesting technique, effectively funnels limited precipitation into the root zone while minimizing non-productive soil evaporation, thereby fostering dry matter accumulation and enhancing water use efficiency (WUE) by approximately 15–20% [19,20,21]. While these water-harvesting techniques possess a historical legacy within traditional Andean agriculture [7], their modern mechanized adaptations have been extensively refined in semi-arid regions of China, particularly for cereal crops [8]. Parallel to this, superabsorbent polymers (SAPs) act as subterranean “micro-reservoirs,” capable of sequestering vast quantities of water and regulating its gradual release during moisture-deficit periods to stabilize the rhizosphere environment [22,23]. Although the discrete advantages of these practices are well-established, their integrated application offers a promising synergistic pathway—extending the photosynthetic duration of leaves and augmenting the overall capacity for carbon assimilation (the “source”).

However, terminal grain yield is not governed solely by the photosynthetic capacity of the “source”; rather, it is contingent upon efficient phloem unloading, long-distance translocation, and strategic partitioning of photoassimilates into the grain (the “sink”). Achieving high productivity thus necessitates a meticulously orchestrated source–sink relationship [24,25,26]. A pivotal scientific gap persists regarding the “over-optimization” of soil hydration: excessive SAP application may trigger luxuriant vegetative growth and delay phenological maturity, thereby exerting an antagonistic effect on reproductive development. This disproportionate allocation of biomass to vegetative organs—primarily stems and leaves—induces a maladaptive “water luxury consumption” and low input efficiency dynamic. Such an imbalance effectively suppresses grain set per unit leaf area and constrains the final harvest index [27,28,29].

Despite the individual merits of ridge-furrow planting and superabsorbent polymers (SAPs), a systematic, quantitative understanding of their combined regulation of quinoa’s source–sink dynamics in semi-arid environments remains elusive. To bridge this critical knowledge gap, this study investigated the interactive effects of these two agronomic strategies. The specific objectives were to: (1) assess the physiological modulation of the photosynthetic “source” under varied planting patterns and SAP rates; (2) quantify the remobilization and translocation efficiency of assimilates to the grain “sink” during reproductive development; and (3) identify the optimal configuration that mitigates vegetative luxury consumption while maximizing source–sink synergy for yield optimization. We hypothesized that ridge-furrow planting coupled with a moderate (60 kg·ha^−1^), rather than maximal, SAP rate would avoid diminishing marginal returns and foster a superior input efficiency, thereby securing sustainable high yields for quinoa in water-limited agroecosystems.

## 2. Results

### 2.1. Effect of Different Treatments on Quinoa Source Performance

#### 2.1.1. Variation Characteristics of Photosynthetic Indicators

**Figure 1:**
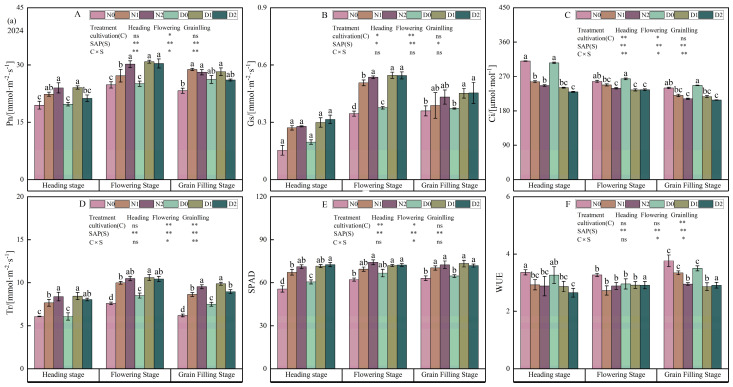

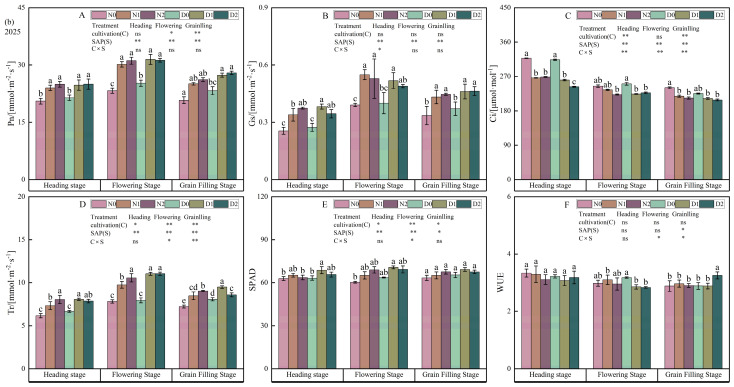
Effects of different planting patterns and superabsorbent polymer (SAP) application rates on the photosynthetic characteristics of quinoa at key reproductive stages in 2024 (**a**) and 2025 (**b**). The sub-panels represent: (A) Net photosynthetic rate (Pn); (B) Stomatal conductance (Gs); (C) Intercellular CO_2_ concentration (Ci); (D) Transpiration rate (Tr); (E) Relative chlorophyll content (SPAD); and (F) Leaf-level water use efficiency (WUE). Treatment abbreviations: N0 (Flat planting + 0 kg ha^−1^ SAP); N1 (Flat planting + 60 kg ha^−1^ SAP); N2 (Flat planting + 90 kg ha^−1^ SAP); D0 (Ridge-furrow planting with furrow sowing + 0 kg ha^−1^ SAP); D1 (Ridge-furrow planting with furrow sowing + 60 kg ha^−1^ SAP); D2 (Ridge-furrow planting with furrow sowing + 90 kg ha^−1^ SAP). Different lowercase letters within the same year indicate significant differences among treatments at *p* < 0.05 (LSD test). C: Planting pattern; S: SAP application rate; C × S: Interaction between C and S. *, ** indicate significant differences at *p* < 0.05 and *p* < 0.01, respectively; ns indicates not significant.

The photosynthetic performance of quinoa exhibited distinct and consistent temporal variations across the three critical reproductive stages over the two-year study (Figure 1a, 2024; Figure 1b, 2025). Generally, the carbon assimilation capacity peaked at the flowering stage before experiencing a physiological decline during the grain-filling stage. The regulatory efficacy of superabsorbent polymers (SAPs) and planting patterns on these physiological traits was highly stage-dependent and reproducible across both years.

Net Photosynthetic Rate (Pn) and Stomatal Conductance (Gs): Analysis of variance (ANOVA) indicated that planting pattern (C), SAP application rate (S), and their interaction (C × S) significantly influenced both Pn and Gs. Specifically, in 2024, the C × S interaction had significant effects on Pn across the three phenological stages (*p* < 0.05 or *p* < 0.01); in 2025, although the interaction effect on Pn was less pronounced, the main factors (C and S) consistently showed highly significant impacts. Similarly, statistical analysis for Gs showed significant impacts from S and C × S interaction in both years, particularly during the heading and grain-filling stages. As illustrated in Figure 1a (Year 2024) and Figure 1b (Year 2024), SAP application significantly enhanced both Pn and Gs across both planting systems. Under flat planting (N), the high SAP rate (N2) consistently maintained superior gas exchange relative to the control (N0). However, the ridge-furrow (D) system demonstrated a clear synergistic advantage, where the D1 treatment (60 kg·ha^−1^ SAP) emerged as the most robust configuration (*p* < 0.05). Notably, during the grain-filling stage in both years, the Pn of D1 remained at peak levels, suggesting that the moderate SAP dose optimally delays leaf senescence and sustains “source” activity. This superior source–sink coordination during the critical period of grain development highlights the mechanistic advantage of combining ridge-furrow planting with moderate SAP application.

Intercellular CO_2_ Concentration (Ci) and Transpiration Rate (Tr): Analysis of variance revealed that planting pattern (C), SAP rate (S), and their interaction (C × S) exerted significant to highly significant influences on both Ci and Tr across both years. Specifically, in 2024 and 2025, the C × S interaction consistently affected Ci and Tr, particularly during the critical flowering and grain-filling stages (*p* < 0.05 or *p* < 0.01). As shown in Figure 1a,b (Panel C, D), Ci displayed a consistent inverse relationship with Pn. The unamended controls (N0 and D0) registered the highest Ci, suggesting that non-stomatal factors or mesophyll limitations potentially constrained carbon assimilation in these groups. Conversely, SAP-treated plants (D1 and N2) exhibited significantly lower Ci, reflecting a more efficient drawdown of internal CO_2_ for carbon assimilation. Concurrently, Tr increased progressively with SAP dosages (Panel D). Although the high transpiration rates observed in D2 and N2 at the flowering stage indicate improved moisture availability, the lack of a proportional gain in Pn—compared to the D1 treatment—suggests that excessive SAP application may trigger “luxury water consumption.” This finding reinforces the D1 configuration as the optimal strategy, which balances the water-saving benefits of SAP with the metabolic demands of quinoa, effectively avoiding the transpiration-driven water waste observed at higher dosage levels.

Relative Chlorophyll Content (SPAD) and Leaf-level Water Use Efficiency (WUE): Analysis of variance (ANOVA) indicated that planting pattern (C), SAP application (S), and their interaction (C × S) exerted significant to highly significant influences on SPAD and leaf-level WUE. Specifically, for SPAD, the C × S interaction consistently exhibited significant effects during the critical flowering stage across both years (*p* < 0.05). For WUE, significant interaction effects were detected during the flowering and grain-filling stages, underscoring the complex dynamic response of quinoa to coordinated soil moisture management. As shown in 2024 and 2025 (Figure 1a,b, Panel E, F), SAP application significantly stimulated chlorophyll biosynthesis, evidenced by the consistently elevated SPAD values in all amended treatments. The D1 and D2 treatments maintained the highest SPAD levels throughout the reproductive phase, indicating that ridge-furrow management effectively sustained the structural integrity of the photosynthetic apparatus. Regarding WUE, a complex trade-off between carbon gain and water loss was observed (Panel F). While SAP application maximized carbon assimilation, it occasionally modulated leaf-level WUE because the increase in Tr (transpiration rate) sometimes outpaced the relative gains in Pn (net photosynthetic rate). Although the N0 treatment exhibited high WUE values, this was largely an artifact of restricted transpiration under water-limited conditions. Crucially, among the high-productivity treatments, D1 maintained a superior balance between carbon gain and water loss compared to the high-dose D2 and N2 treatments. By avoiding the WUE penalties associated with excessive transpiration, D1 demonstrated an optimized physiological state for sustaining reproductive growth, further reinforcing its role in harmonizing the source–sink relationship.

#### 2.1.2. Variation Characteristics of Quinoa Leaf Area Index (LAI)

**Figure 2:**
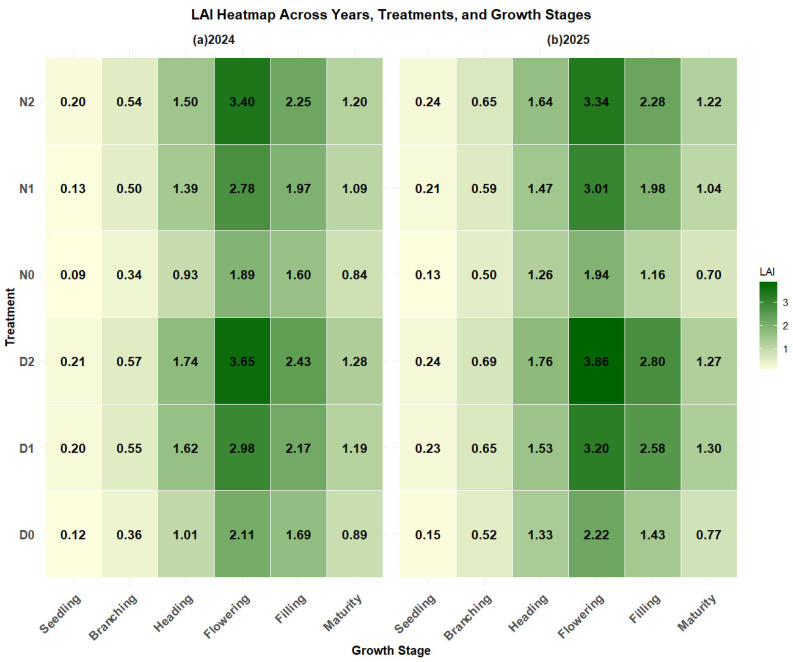
Spatiotemporal dynamics and hierarchical clustering of quinoa Leaf Area Index (LAI) under different planting patterns and superabsorbent polymer (SAP) application rates in (**a**) 2024 and (**b**) 2025. Treatment abbreviations: N0 (Flat planting + 0 kg ha^−1^ SAP); N1 (Flat planting + 60 kg ha^−1^ SAP); N2 (Flat planting + 90 kg ha^−1^ SAP); D0 (Ridge-furrow planting with furrow sowing + 0 kg ha^−1^ SAP); D1 (Ridge-furrow planting with furrow sowing + 60 kg ha^−1^ SAP); D2 (Ridge-furrow planting with furrow sowing + 90 kg ha^−1^ SAP).

Figure 2 illustrates the dynamic variations in the Leaf Area Index (LAI) of quinoa across six phenological stages (seedling, branching, heading, flowering, grain-filling, and maturity) under different treatments over two consecutive years (2024 and 2025). The analysis reveals that ridge-furrow planting consistently enhanced canopy development throughout the growing season, with the most pronounced advantages manifesting from the flowering stage through to maturity. Irrespective of the specific treatments, the temporal dynamics of LAI exhibited a highly reproducible pattern across both years: values consistently peaked at the flowering stage (reaching a two-year average of 3.03) prior to experiencing a gradual, senescence-driven decline. Furthermore, canopy expansion exhibited a positive dose–response relationship with the SAP application rate. Notably, under the medium and high SAP doses, the LAI in the ridge-furrow system was 7.29% and 10.59% higher, respectively, than that in the corresponding flat planting system, underscoring a substantial advantage in vegetative structural development. Specifically, the D1 treatment (ridge-furrow + 60 kg·ha^−1^ SAP) increased the whole-season average LAI by 31.25% and the peak LAI at flowering by a remarkable 42.40% relative to the unamended control (D0). Moreover, D1 outperformed its flat-planting counterpart (N1) by 8.11%. Crucially, the D1 configuration successfully established an optimal canopy architecture, effectively avoiding the “water luxury consumption” and severe diminishing marginal returns associated with the massive vegetative canopy observed in the high-SAP treatment (D2). This demonstrates that D1 provides a highly robust photosynthetic source while maximizing agronomic input efficiency.

#### 2.1.3. Dry Matter Accumulation and Partitioning

**Figure 3:**
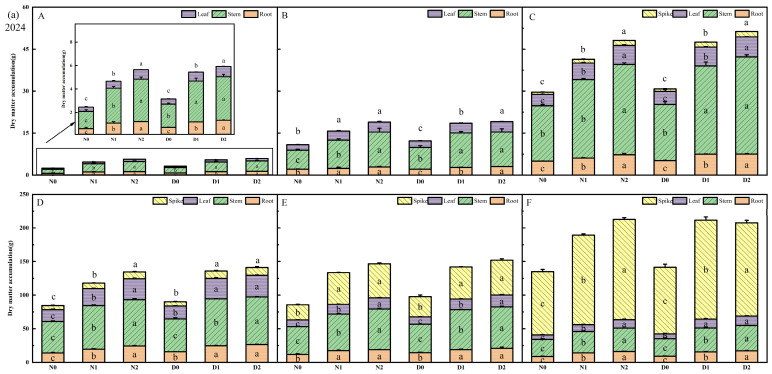

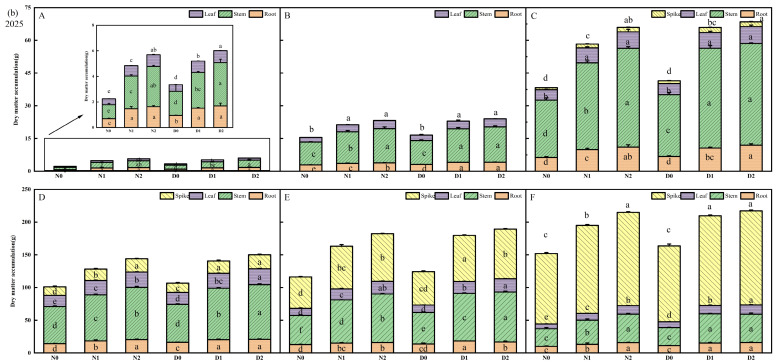
Dynamic accumulation and partitioning of dry matter in different quinoa organs (root, stem, leaf, and panicle) at key phenological stages in (**a**) 2024 and (**b**) 2025. The sub-panels represent the corresponding growth stages: (A) Seedling stage; (B) Branching stage; (C) Heading stage; (D) Flowering stage; (E) Grain-filling stage; and (F) Maturity stage. Treatment abbreviations: N0 (Flat planting + 0 kg ha^−1^ SAP); N1 (Flat planting + 60 kg ha^−1^ SAP); N2 (Flat planting + 90 kg ha^−1^ SAP); D0 (Ridge-furrow planting with furrow sowing + 0 kg ha^−1^ SAP); D1 (Ridge-furrow planting with furrow sowing + 60 kg ha^−1^ SAP); D2 (Ridge-furrow planting with furrow sowing + 90 kg ha^−1^ SAP). Different lowercase letters within the same year indicate significant differences among treatments at *p* < 0.05 (LSD test).

The accumulation and partitioning of biomass are integral indicators that reflect the interplay between photosynthetic “source” capacity and reproductive “sink” activity, directly dictating final yield formation. This study systematically analyzed the temporal dynamics of dry matter accumulation in various organs (roots, stems, leaves, and panicles) of quinoa across key phenological stages from 2024 to 2025, with the comprehensive results presented in Figure 3. Over the two-year study, the dynamic patterns of biomass accumulation were highly consistent. As the growth progressed, total plant dry matter exhibited a continuous upward trajectory (Figure 3 panels A–C). During the vegetative phase, biomass accumulation was minimal, with roots, stems, and leaves acting as the primary partitioning sinks. Entering the flowering stage, dry matter accumulation accelerated rapidly, accompanied by a progressive increase in the allocation ratio to the panicles. By maturity (Figure 3 panel F), total dry matter reached its apex for the entire growth cycle, and the panicle was established as the primary sink for assimilate partitioning. This progression aligns perfectly with the classic quinoa growth paradigm of “early source establishment, followed by late-season remobilization and grain filling. Regarding planting patterns, ridge-furrow planting (D) not only enhanced vegetative biomass accumulation but also optimized assimilate partitioning during the reproductive phase. Across the 2024–2025 seasons, the total dry matter at the flowering stage under the D1 and D2 treatments increased by an average of 11.2% and 8.7%, respectively, compared to their flat-planting counterparts (N1 and N2). This trend is in strong agreement with the observed enhancements in net photosynthetic rate (Pn) and leaf area index (LAI), verifying that ridge-furrow planting optimizes the root-zone microenvironment to expand “source” size while simultaneously boosting biomass production efficiency. Crucially, at maturity, the proportion of dry matter allocated to vegetative organs (roots and stems) in the ridge-furrow treatments was significantly lower than in the flat planting treatments, whereas the panicle biomass ratio and assimilate translocation efficiency were significantly higher in 2024 and 2025 (Figure 3 panels E,F). This demonstrates the sink-enhancing advantage of ridge-furrow planting in suppressing redundant vegetative growth and accelerating the remobilization of stored photoassimilates to the grain sink, laying a solid physiological foundation for source–sink synergy and yield improvement.

From the perspective of SAP dosage, increasing SAP application significantly elevated total dry matter accumulation across all stages. The high-dose SAP treatments (N2 and D2) increased total dry matter at maturity by an average of 78.3–82.5% compared to the unamended control (N0 and D0). However, excessive SAP application (N2 and D2) induced a severe imbalance in dry matter partitioning. The overly vigorous early vegetative growth led to excessive biomass sequestration in vegetative organs, resulting in canopy overgrowth. Consequently, at maturity, the proportion of biomass retained in stems and leaves under N2 and D2 was significantly higher than in the medium SAP treatment (N1 and D1), while the partitioning efficiency to the panicles dropped sharply. This culminated in a pronounced “water luxury consumption” and low input efficiency phenotype, ultimately compromising the grain-to-leaf ratio and final yield potential.

#### 2.1.4. Dynamic Variation in Crop Growth Rate (CGR)

Crop growth rate (CGR), defined as the dry matter accumulated per unit land area per unit time, serves as a core physiological indicator for quantifying photosynthetic production efficiency. As presented in Table 1, ANOVA indicated that SAP application (S) exerted highly significant effects on CGR across all phenological stages (*p* < 0.01), while the planting pattern (C) and the interaction (C × S) showed stage-specific significance.

Over the two-year study, CGR exhibited a consistent dynamic pattern for all treatments, characterized by an initial increase followed by a subsequent decline. The maximum CGR occurred during the heading-to-flowering window, coinciding with peak LAI, which underscores the critical importance of resource supply during this period for establishing a robust photosynthetic “source.” Regarding planting patterns, ridge-furrow planting (D) significantly accelerated CGR during the early vegetative stages (Table 1). Specifically, the D1 and D2 treatments increased CGR during the branching-to-heading stage by an average of 16.3% and 6.2%, respectively, compared to flat-planting (N1 and N2). This aligns with the observed enhancements in Pn and LAI, validating that this micro-topographic modification optimizes the root-zone environment to expand canopy scale.

From the perspective of SAP dosage, increasing SAP application significantly elevated CGR from sowing to flowering (*p* < 0.01). However, a highly significant main effect of SAP application (S) was observed during the late grain-filling-to-maturity stage, where high SAP treatments (N2 and D2) showed a precipitous decline in CGR, averaging 23.1% lower than the unamended control. This pattern corroborates our source–sink analysis: while a moderate SAP dose (D1) promotes early vegetative growth to build a strong “source,” excessive SAP application triggers overly vigorous early growth and delayed senescence. This eventually compromises the efficiency of dry matter accumulation during the critical grain-filling phase, manifesting as a typical “water luxury consumption” and low input efficiency maladaptation.

### 2.2. Effects of Different Treatments on Quinoa Sink Performance and Yield

Sink capacity and activity are the fundamental determinants of photoassimilate partitioning and ultimate grain productivity. To systematically elucidate the regulatory effects of planting patterns and SAP application rates on quinoa sink performance, this study performed a multi-dimensional analysis of grain weight per plant (GWPP), thousand-kernel weight (TKW), and total grain yield (Y) across the 2024 and 2025 investigated vegetation periods. Analysis of variance (ANOVA) indicated that planting pattern (C) and SAP application rate (S) exerted significant to highly significant impacts on Y, TKW, and GWPP across both study years (*p* < 0.05 or *p* < 0.01, Table 2). Notably, while the interaction (C × S) did not significantly affect Y or GWPP, it demonstrated a highly significant impact on TKW in 2024, suggesting that the coordination of planting geometry and SAP is critical for grain-filling quality in certain climatic contexts.

**Figure 4:**
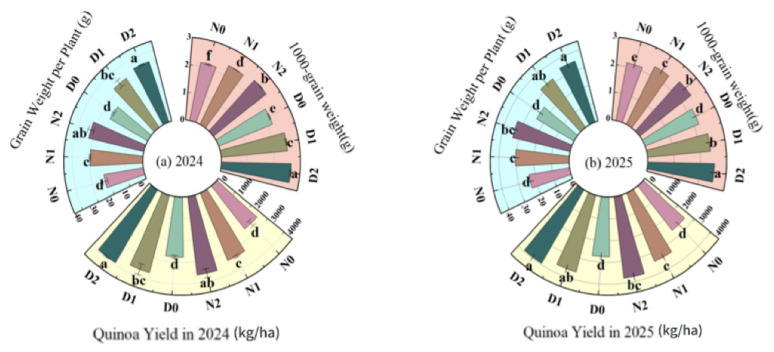
Effects of different planting patterns and superabsorbent polymer (SAP) application rates on quinoa sink performance and yield components in 2024 and 2025: Values followed by different letters within the column are significantly different at *p* < 0.05. Treatment abbreviations: N0 (Flat planting + 0 kg ha^−1^ SAP); N1 (Flat planting + 60 kg ha^−1^ SAP); N2 (Flat planting + 90 kg ha^−1^ SAP); D0 (Ridge-furrow planting with furrow sowing + 0 kg ha^−1^ SAP); D1 (Ridge-furrow planting with furrow sowing + 60 kg ha^−1^ SAP); D2 (Ridge-furrow planting with furrow sowing + 90 kg ha^−1^ SAP).

As illustrated in Figure 4, the ridge-furrow (D) system consistently outperformed flat planting (N) across all sink-related metrics. Furthermore, as the SAP application rate increased, total grain yield and sink quality indicators continued to rise, reaching their maximum absolute values under the high-dose treatments (N2 and D2). However, from an agronomic application and input efficiency perspective, the D1 treatment (60 kg ha^−1^ SAP) consistently emerged as the optimal configuration for balancing sink performance and actual yield. For instance, based on the two-year average, compared to the unamended control (N0), D1 significantly increased Y and TKW by 38.12% and 12.99%, respectively.

A crucial agronomic finding is the diminishing marginal returns identified in high-dose treatments (N2 and D2). Although these treatments achieved the highest absolute TKW and yield, the marginal agronomic benefit diminished considerably. Increasing the SAP dosage by 50% (from 60 kg ha^−1^ to 90 kg ha^−1^) only resulted in a disproportionately small yield gain. This indicates a low input efficiency under excessive moisture management. Conversely, unamended controls (N0 and D0) suffered from persistent water stress, manifesting a characteristic “weak source, small sink” phenotype.

Mechanistically, the ridge-furrow system functions as a dynamic regulator of root-zone moisture; it imposes a moderate and effective regulation during the late reproductive stages, accelerating the unloading of photoassimilates from storage organs (stems/leaves) to the developing grains. By maintaining a robust canopy “source” while optimizing “sink” activity, the D1 configuration achieves a superior harvest index. This integration of micro-topographic modification and precise moderate SAP modulation demonstrates a sophisticated strategy for harmonizing assimilate partitioning, providing the ultimate explanation for why D1 represents the best balance between productivity and resource input.

### 2.3. Responses of the Quinoa Sink-Source Ratio to Different Treatments

Source and sink organs in crops are mutually constrained, and a highly coordinated relationship between them is a fundamental prerequisite for maximizing yield. The sink-source ratio serves as a critical quantitative metric for evaluating this dynamic canopy-level coordination. In agronomic practice, breaking through existing yield ceilings under a constrained maximum leaf area necessitates steadily increasing the sink capacity (grain load) per unit of photosynthetic source. Analysis of variance (ANOVA) indicated that the SAP application rate (S) exerted a highly significant influence on the grain weight-to-LAI ratio and grain number-to-leaf dry weight ratio across both years (*p* < 0.01, Table 3). While the planting pattern (C) significantly affected the grain weight-to-LAI ratio in 2024, the interaction (C × S) showed stage-specific significance for the grain number-to-LAI ratio in 2025.

Table 3 details the population-level grain-to-leaf ratio of quinoa under various treatments. Regardless of the planting geometry—ridge-furrow (D) or flat (N)—the sink-source ratio exhibited a highly significant inverse relationship with the SAP application rate across both years. Based on two-year averages, under flat planting, the grain weight-to-LAI ratio in the unamended control (N0) was 68.0% and 73.0% higher than in the N1 and N2 treatments, respectively. Similarly, under the ridge-furrow system, the ratio in D0 surpassed those of D1 and D2 by 42.7% and 56.6%, respectively.

This phenomenon is strictly governed by the physiological principles of source–sink allometry. While SAP application significantly ameliorates soil moisture and drives exponential canopy leaf area expansion, the concomitant gains in grain yield are disproportionately smaller. Consequently, the grain load per unit of photosynthetic source (represented by leaf area or leaf dry weight) inevitably declines as SAP dosage increases.

Notably, the D1 treatment (ridge-furrow + 60 kg·ha^−1^ SAP) achieved an optimal physiological compromise. While it significantly enhanced canopy LAI (+31.25% relative to D0) and population photosynthetic capacity, it effectively mitigated the severe reduction in the sink-source ratio observed under high SAP inputs. Specifically, based on two-year averages, D1 maintained a 9.7% higher grain weight-to-LAI ratio and an 18.9% higher grain number-to-LAI ratio compared to the excessive SAP treatment (D2). This indicates that the D1 configuration successfully circumvents the canopy mutual shading and subsequent source–sink decoupling induced by luxury moisture supply. Ultimately, it realizes an ideal agronomic state: “an adequate canopy source coupled with high production efficiency per unit source.”

Furthermore, when compared to its flat-planting equivalent (N1), the D1 treatment exhibited a nearly identical sink-source ratio but possessed a markedly superior LAI, net photosynthetic rate, and Leaf instantaneous water use efficiency. This demonstrates that D1 provides a vastly larger canopy photosynthetic production potential without sacrificing the degree of source–sink coordination. Therefore, integrating ridge-furrow planting with a moderate SAP application is the definitive strategy for harmonizing robust population vegetative growth with optimal reproductive partitioning.

### 2.4. Principal Component Analysis of Source–Sink Performance and Yield

To systematically unravel the multivariate interplay among superabsorbent polymers (SAPs), planting geometry, photosynthetic physiology, and yield components, a Principal Component Analysis (PCA) was conducted using 10 core evaluation metrics. The first two principal components (PCs) captured a cumulative explained variance of 85.0% (PC1: 73.4%; PC2: 11.6%). This robust extraction sufficiently represents the core data structure, providing a comprehensive evaluation of source–sink coordination and yield formation under varied agronomic interventions. PC1 was primarily driven by positive loadings from net photosynthetic rate (Pn), stomatal conductance (Gs), transpiration rate (Tr), relative chlorophyll content (SPAD), leaf area index (LAI), total dry matter (Dry), grain yield (GY), and thousand-grain weight (TKW). Biologically, PC1 can be defined as the “Canopy Photosynthetic Production and Biomass Dimension,” reflecting the absolute strength of the crop’s photosynthetic source and sink capacity. Conversely, PC2 was primarily driven by positive loadings from leaf instantaneous water use efficiency (WUE) and negative loadings from transpiration rate (Tr), with Ci acting as a major negative contributor to PC1. Thus, PC2 was designated as the “Water Utilization and Resource Efficiency Dimension.” In the PCA ordination space, the treatment clusters exhibited distinct and biologically meaningful trajectories. The unamended controls (N0, D0) localized in the upper-left quadrant (negative PC1, positive PC2), typifying a “low production, high WUE” survival strategy under water deficit. With increasing SAP rates, treatments migrated significantly to the right along the PC1 axis, denoting synchronous enhancements in photosynthesis and absolute yield traits. However, while high-SAP treatments (N2, D2) scored highest on PC1 (maximizing absolute production), they exhibited a marked downward shift along the PC2 axis. Since instantaneous WUE is determined by the ratio of Pn to Tr, this spatial divergence mathematically substantiates that excessive moisture induces “water luxury consumption.” Specifically, the high SAP inputs drive up the transpiration rate (Tr) excessively without proportional gains in yield, thereby significantly compromising water use efficiency. In stark contrast, the D1 treatment (ridge-furrow + 60 kg·ha^−1^ SAP) was stably positioned in the core of the first quadrant (high PC1, high PC2), signifying a dual mastery of powerful source manufacturing capacity and optimal resource conservation. Analysis of the variable vectors (loadings) revealed that most “source” (Pn, Gs, SPAD, Dry, LAI) and “sink” (GY, TKW) indicators clustered tightly on the positive PC1 axis with acute angles between them. This geometric proximity confirms a highly significant positive correlation, corroborating that a robust photosynthetic source is the fundamental prerequisite for yield improvement. Interestingly, WUE (positive PC2) lacked a strong acute-angle alignment with absolute yield indicators, implying an inherent physiological trade-off between maximizing absolute production and maximizing water conservation—a conflict that necessitates optimized agronomic interventions to resolve. Within the treatment matrix, D2 localized at the extreme right of PC1 but suffered significantly on PC2, indicating diminished marginal returns regarding water and SAP inputs. Conversely, the D1 treatment achieved the optimal coordinates. By maintaining high-tier source strength (PC1) while significantly avoiding the severe drop in WUE seen in high-SAP treatments (PC2), D1 visually and statistically proves to be the optimal management paradigm for harmonizing high productivity with superior input efficiency in semi-arid quinoa cultivation.

## 3. Discussion

### 3.1. Synergistic Effects of Ridge-Furrow Planting and SAPs on Quinoa Photosynthetic “Source” and WUE

Crop productivity fundamentally relies on dry matter accumulation, which is driven by photosynthetic “source” strength [24]. Our results demonstrated that the integration of ridge-furrow planting and SAP application significantly enhanced quinoa’s photosynthetic performance, characterized by increased Pn, Gs, and SPAD, alongside an optimized Ci. These findings align with previous research on other semi-arid grain crops, such as maize and wheat, where the optimization of root-zone micro-environments has been identified as a critical driver for yield potential [20,21].

**Figure 5:**
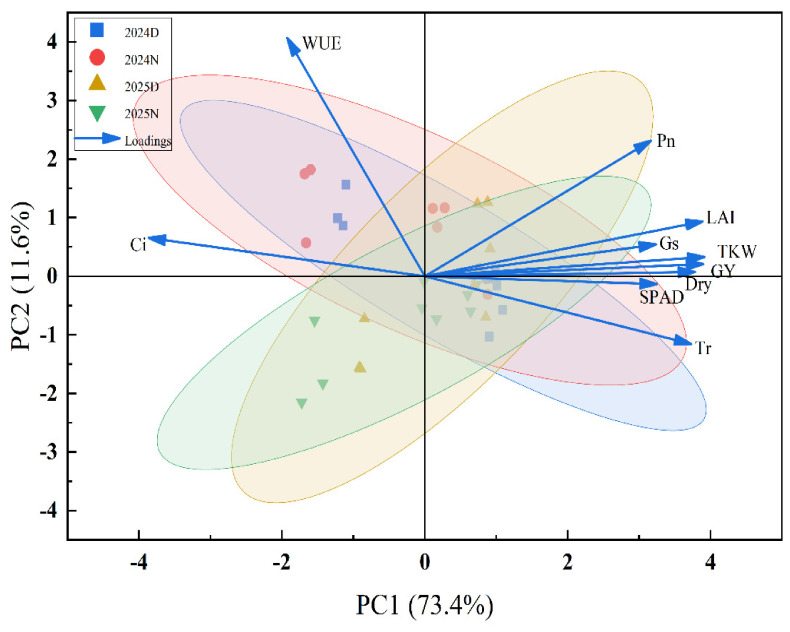
Principal component analysis (PCA) biplot illustrating the relationships among photosynthetic source performance, sink capacity, and water use efficiency under different treatments in 2024 and 2025. Notes: The vectors (blue arrows) represent the physiological and yield variables, while the scattered points represent the individual treatment replicates. PC1 and PC2 account for 73.4% and 11.6% of the total variance, respectively. Abbreviations: *P*n, net photosynthetic rate; Gs, stomatal conductance; *C*i, intercellular CO_2_ concentration; *T*r, transpiration rate; SPAD, relative chlorophyll content; LAI, leaf area index; Dry, total dry matter accumulation; GY, grain yield; TKW, thousand-kernel weight; WUE, water use efficiency. Treatment abbreviations: N0 (Flat planting + 0 kg ha^−1^ SAP); N1 (Flat planting + 60 kg ha^−1^ SAP); N2 (Flat planting + 90 kg ha^−1^ SAP); D0 (Ridge-furrow planting with furrow sowing + 0 kg ha^−1^ SAP); D1 (Ridge-furrow planting with furrow sowing + 60 kg ha^−1^ SAP); D2 (Ridge-furrow planting with furrow sowing + 90 kg ha^−1^ SAP).

This pronounced physiological improvement is primarily driven by the synergistic stabilization of soil moisture. The ridge-furrow geometry functions as a “spatial rainwater harvesting” mechanism, while SAPs operate as subterranean “mini-reservoirs” that regulate moisture availability during dry spells [22,23]. This dual-buffer strategy effectively mitigates drought-induced damage to the photosynthetic apparatus and sustains canopy longevity. Crucially, these physiological gains are not isolated; they serve as the direct material foundation for grain yield formation. By maintaining a robust photosynthetic “source” during the reproductive phase, this management strategy facilitates the accumulation and remobilization of photoassimilates, which significantly contributed to the increased thousand-kernel weight (TKW) and total grain yield observed in our study (Figure 5).

Compared to high-dosage SAP treatments (e.g., 90 kg·ha^−1^) that may trigger “water luxury consumption” and excessive transpiration without proportional gains in grain yield, the moderate SAP rate (60 kg·ha^−1^) achieves an optimal agronomic balance. Our results confirm that the coupling of ridge-furrow planting (D) and moderate SAP dosage is a high-efficiency agronomic pathway that harmonizes robust population vegetative growth with optimal resource utilization, thereby stabilizing grain yield in water-limited semi-arid environments [11,12,17,30,31].

### 3.2. Regulatory Effects of Planting Patterns and SAP Dosage on Sink Performance

The capacity and activity of the “sink” determine the efficiency of photoassimilate partitioning and storage [26]. Crop yield depends not only on “source” production but also on the quality of “sink” filling [24,29]. In this study, SAP application significantly increased final yield, grain weight per plant, and thousand-grain weight (TKW). However, a critical observation was made: as SAP application rates increased to the highest level, the marginal agronomic benefit diminished considerably. While the D2 treatment achieved the highest absolute yield and TKW, the disproportionate ratio between the massive canopy size and the grain output revealed a limitation of excessive SAP application in semi-arid regions. Our PCA results further supported this. The high-dose SAP treatments (N2 and D2) positioned treatments far to the right on PC1 (high productivity). However, it showed a clear decline on the PC2 axis (water utilization and resource efficiency). This separation quantitatively demonstrates that “maximal absolute production” does not equate to “efficient resource use.” Excessive moisture input easily induces luxuriant early vegetative growth and excessive transpiration, which we termed “water luxury consumption.” This diverts massive amounts of soil moisture without delivering a proportional increase in grain yield [25]. Mathematically, this manifests as a deviation in the developmental trajectories of PC1 and PC2. Ridge-furrow planting, under dryland conditions, helps mitigate this by optimizing root-zone resources towards the grains, enhancing sink activity without wasteful vegetative redundancy [10].

From an economic perspective, while the implementation of ridge-furrow planting and SAP application entails higher initial operational costs compared to conventional flat planting, this investment is strategically justified by the yield-stabilizing effect under semi-arid conditions. In regions where drought-induced crop failure is a significant threat, the D1 configuration (ridge-furrow + 60 kg·ha^−1^ SAP) minimizes the risk of production loss and optimizes the yield-input ratio. Our findings suggest that this synergistic management strategy represents a cost-effective pathway, as the substantial enhancement in grain yield and quality significantly offsets the moderate additional input costs. Future large-scale agricultural extension will include a comprehensive cost–benefit analysis to further refine these input parameters for local farmers. Specifically, our analysis indicates that while the D1 treatment involves moderate input costs, the yield gain (an average 60.3–67.9% increase in total yield compared to the control) provides a far superior return on investment than the high-input D2 treatment, where a 50% increase in SAP dosage only results in marginal yield gains.

### 3.3. Key Factor Analysis: Mechanisms Driving Source–Sink Coordination and Yield

Sustainable agriculture in semi-arid regions must balance yield, efficiency, and cost [1,3,5]. Simply increasing SAP application to create a “large source” does not guarantee a highly efficient agronomic system. Instead, excessive inputs may cause resource waste [2]. High practical productivity fundamentally depends on strong source–sink coordination and input efficiency [27].

Our PCA results revealed the driving mechanisms of yield formation. Quinoa yield is highly and positively correlated with dry matter accumulation, LAI, chlorophyll content, and photosynthetic rate. This proves that a powerful “source” is a prerequisite for high yield. In complex semi-arid ecosystems, single indicators often have limitations. Through PCA, we found that PC1 represents the absolute “Canopy Photosynthetic Production.” While PC1 is the primary driver of absolute yield, the most significant finding of this study involves PC2, which governs “Resource Efficiency and Water Utilization.” When SAP supply is excessive (e.g., N2 and D2), the growth trajectory deviates from the optimal quadrant. It shows maximum absolute production on the PC1 axis but a severe efficiency drop on the PC2 axis due to excessive transpiration. This spatial distribution suggests a physiological imbalance: excessive SAP creates an overly massive canopy that demands immense water resources, leading to diminished marginal returns. The PCA biplot clearly shows differences in the “ecological niches” of various treatments. The D1 treatment (ridge-furrow + 60 kg·ha^−1^ SAP) is stably located in the core of the first quadrant. It scores highly on both PC1 (source strength and yield) and PC2 (water and input efficiency).

Therefore, while the D2 treatment achieves the highest absolute yield, the D1 treatment appears to provide the best balance between productivity and input efficiency. Considering water use efficiency, resource allocation, and the principle of diminishing marginal returns, ridge-furrow planting with 60 kg·ha^−1^ SAP emerges as the most robust agronomic strategy under the conditions of this study. This configuration effectively harmonizes the source–sink relationship and avoids resource waste, thereby ensuring highly efficient and sustainable quinoa production in semi-arid regions [10,15].

## 4. Materials and Methods

### 4.1. Site Description

**Figure 6:**
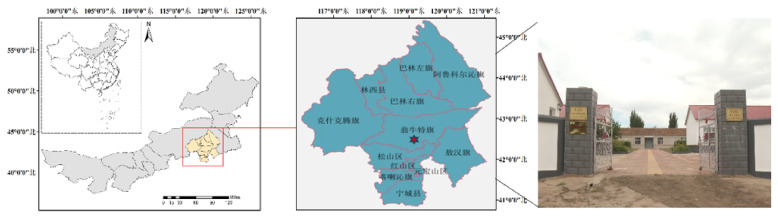
Location map of the sampling site in the study area.

**Figure 7:**
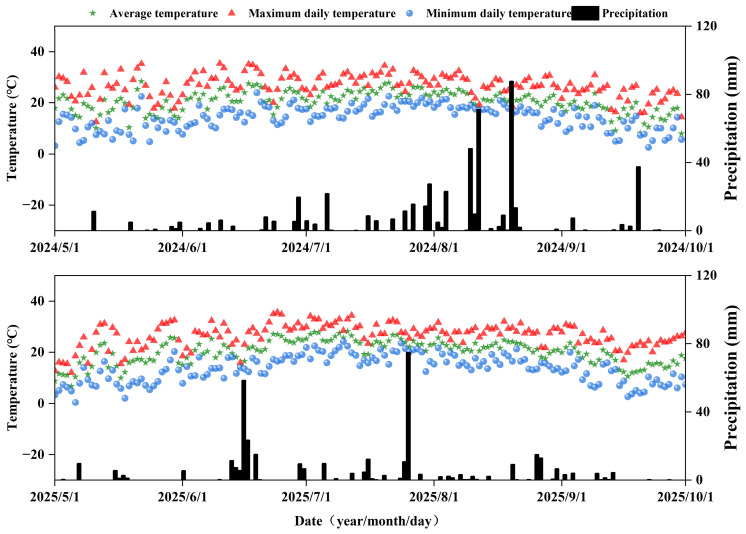
Daily precipitation and maximum, mean, and minimum air temperatures during the investigated vegetation periods.

The research site is located at the Quinoa Science and Technology Center in Wengni-ute Banner, Chifeng City, Inner Mongolia Autonomous Region, China (119.48° E, 42.83° N) (Figure 6). The region belongs to the Yanshan hilly area and features a mid-temperate semi-arid continental monsoon climate. The average annual temperature is 6.4 °C, and the average annual precipitation is approximately 370 mm, with rainfall concentrated between July and August, and the frost-free period lasts around 135 days. Meteorological data during the investigated vegetation periods in 2024–2025 are shown in Figure 7. The soil type is Kastanozem (FAO/WRB classification), and the basic soil properties in the 0–20 cm layer (representing pre-sowing samplings in 2024) are: soil organic matter (SOM) = 15.33 g kg^−1^, ammoniacal nitrogen = 10.38 mg kg^−1^, nitrate nitrogen = 2.66 mg kg^−1^, available phosphorus = 13.41 mg kg^−1^, and available potassium = 149.12 mg kg^−1^.

### 4.2. Experimental Design

**Figure 8:**
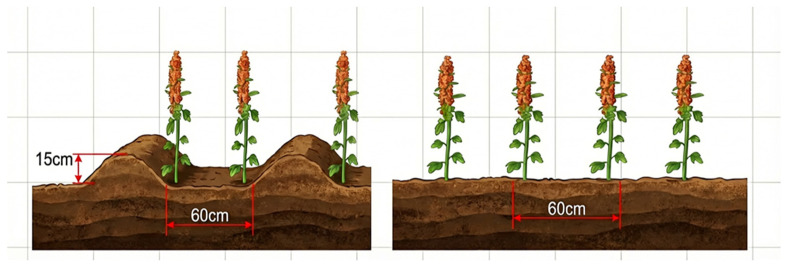
The planting pattern is shown.

The studied quinoa material was ‘Longli 5’, a stabilized elite genotype (cultivar) developed through selection by the Gansu Academy of Agricultural Sciences, China. This cultivar features high agronomic stability and is well-adapted to the semi-arid environmental conditions of the region. Two planting patterns were adopted in the experiment: flat planting (N) and ridge-furrow planting with furrow sowing (D). A split-plot design was used, with the main plots representing the planting patterns and the subplots representing the application rates of the superabsorbent polymer (SAP). The SAP used in this study was a cross-linked polyacrylamide (PAM) based water-retaining agent (Zhidong Zaiti, purchased from Beijing Hanlimiao New Technology Co., Ltd., Beijing, China). The SAP application rates were set at three levels: N0 and D0(0 kg ha^−1^), N1 and D1(60 kg ha^−1^), and N2 and D2(90 kg ha^−1^). Consequently, a total of six treatments were established: N0, N1, and N2 (flat planting combined with 0, 60, and 90 kg ha^−1^ SAP, respectively) and D0, D1, and D2 (ridge-furrow planting with furrow sowing combined with 0, 60, and 90 kg ha^−1^ SAP, respectively). Each treatment was replicated three times. The plot size was 4 m × 6 m = 24 m^2^ (with a 1 m interval between plots). Urea (N ≥ 46%), superphosphate (P_2_O_5_ ≥ 18%), and potassium sulfate (K_2_O ≥ 52%) were used as fertilizers at rates of 82.8 kg ha^−1^, 105 kg ha^−1^, and 60 kg ha^−1^, respectively. All phosphorus and potassium fertilizers, along with 50% of the nitrogen fertilizer, were applied as base fertilizers in a single application and incorporated into the 0–15 cm soil layer by a rotary tiller before sowing. The remaining 50% of the nitrogen fertilizer was applied as top-dressing at the jointing stage. Sowing was carried out by mechanical furrowing followed by manual seeding in the middle of the furrows. Other agronomic management measures were consistent with the local conventional practices. Sowing was conducted in early May each year, and harvesting was completed by the end of September. The planting pattern is shown in Figure 8.

### 4.3. Measurement of the Leaf Photosynthetic Characteristics

At the beginning of the quinoa booting stage, uniform plants within each plot were selected, tagged, and marked. During the flowering stage, nine quinoa plants were chosen to measure gas exchange, chlorophyll content (SPAD value), and leaf area index of the leaves at the main panicle position. Measurements were conducted on clear days between 9:00 and 11:00 a.m. Net photosynthetic rate (Pn), stomatal conductance (Gs), intercellular CO_2_ concentration (Ci), and transpiration rate (Tr) were determined using the CIRAS-3 portable photosynthesis instrument is produced by the American company PP-System (Amesbury, MA, USA). The measurement conditions were as follows: equipped with a 2 cm^2^ leaf chamber, flow rate set at 500 μmol s^−1^, photosynthetic photon flux density maintained at 1200 μmol m^−2^ s^−1^, leaf temperature kept consistent with ambient air temperature, relative humidity stabilized at 55%, and measurements taken at the middle section of the leaf while avoiding the midrib and leaf margin. At the same time, nine labeled leaves in each plot were chosen to measure the chlorophyll content (SPAD) with a Minolta SPAD-502 chlorophyll meter (Minolta, Japan). The measurement was done three times for each leaf, and the mean was calculated as the SPAD value of the given leaf. Subsequently, the leaf area per plant was determined using the specific leaf weight method, which was then used to calculate the leaf area index (LAI). Furthermore, the leaf instantaneous water use efficiency (WUE) was calculated as the ratio of net photosynthetic rate (Pn) to transpiration rate (Tr).

Leaf instantaneous water use efficiency (WUE) = Pn/Tr(1)

Leaf area index = leaf area per plant (m^2^ plant^−1^) × plant density (plants m^−2^) (2)

### 4.4. Measurement of the Agronomic Traits and Grain Yield

Dry matter content: In each plot during the seedling stage, branching stage, ear emergence stage, flowering stage, grain filling stage and maturity stage, 6 quinoa plants were randomly selected. After blanching at 105 °C for 30 min and adjusting the oven temperature to 80 °C to dry until a constant weight was achieved, the dry weight was weighed and converted to the dry matter accumulation per hectare based on the planting density. The population growth rate (CGR) was calculated using the following formula:

CGR = (WS2 − WS1)/(TS2 − TS1)(3)

where CGR represents the population growth rate from TS2 to TS1 stage (kg/hm^2^/d), TS2 to TS1 is the measurement period, WS2 and WS1 are the dry matter weights of TS2 and TS1 periods (kg/hm^2^), and the measurement period is the sampling date of the above quinoa growth stages.

During the quinoa maturity stage, 10 intact and uniformly growing plants were selected from each plot to measure grain yield (GY) and 1000-grain weight (TKW). The final grain yield was standardized at 14.0% moisture content. The grain yield presented in this study refers to the actual yield of quinoa after considering the lodging loss.

### 4.5. Statistical Analysis

Statistical analysis was performed using SPSS software version 27.0 (IBM Corporation, Version 27.0, Armonk, NY, USA). The experimental results were statistically analyzed using the least significant difference test (*p* < 0.05, LSD 0.05). In this study, two-factor analysis of variance was used to explore the effects of planting methods and water-retaining agent application rates on various indicators. All data were plotted using Origin 2025 software. Principal component analysis was conducted using this software to determine the relationship between quinoa yield and source and sink performance.

## 5. Conclusions

This two-year field experiment indicates that the integration of ridge-furrow planting with a moderate superabsorbent polymer (SAP) application (60 kg·ha^−1^) appears to be a high-efficiency strategy for optimizing quinoa productivity under the conditions of this semi-arid region. Using the genotype ‘Longli 5’, our results reveal that this integrated management system may function as a multi-scale regulatory mechanism for yield formation.

Physiologically, the ridge-furrow and SAP synergy functions as a “subterranean reservoir,” alleviating seasonal water-demand mismatches. This modulation optimizes source–sink coordination by expanding the canopy photosynthetic “source” (LAI) and accelerating the remobilization of photoassimilates to the “sink” (grains). Crucially, this strategy translates physiological improvements into tangible yield benefits: based on the two-year average, the D1 treatment significantly increased grain yield (Y) and thousand-kernel weight (TKW) by 38.12% and 12.99%, respectively, compared to the unamended control (N0). In contrast, higher SAP rates (90 kg·ha^−1^) induced “luxury water consumption”, leading to diminishing marginal returns. Therefore, a moderate SAP rate (60 kg·ha^−1^) achieves the optimal balance between absolute productivity and input efficiency. This study provides a mechanistic framework for modernizing rainfed quinoa cultivation, offering a scalable agronomic solution that stabilizes food production and optimizes resource inputs for quinoa growers in arid and semi-arid environments.

## Figures and Tables

**Table 1:** Dynamic variations in crop growth rate (CGR, g·m^−2^·d^−1^) of quinoa at different growth stages under various planting patterns and superabsorbent polymer (SAP) application rates in 2024 and 2025.

Years	Treatments	Seedling- Branching	Branching- Heading	Heading- Flowering	Flowering- Grain Filling	Grain Filling- Maturity
2024	N0	6.23 d	14.04 e	31.54 c	11.61 e	20.98 a
	N1	8.10 b	19.36 c	42.44 a	17.44 abc	17.35 abc
	N2	9.28 a	22.56 b	43.66 a	19.97 ab	15.26 c
	D0	6.72 c	13.91 e	34.78 b	11.96 de	19.97 abc
	D1	9.30 a	22.58 b	43.72 a	16.55 bcd	16.28 abc
	D2	9.54 a	24.44 a	43.55 a	21.33 ab	16.19 abc
Variation source						
Cultivate (C)		**	**	ns	ns	ns
SAP(S)		**	**	**	**	**
C × S		ns	**	ns	ns	ns
2025	N0	7.41 e	12.94 e	35.36 d	6.79 d	20.08 ab
	N1	9.21 bc	20.98 b	39.36 c	15.74 ab	17.79 bc
	N2	9.79 ab	24.21 a	43.94 ab	17.21 a	18.35 bc
	D0	7.37 e	14.13 e	36.66 d	7.89 d	22.22 a
	D1	9.93 ab	24.32 a	42.01 b	17.62 a	16.85 bc
	D2	10.09 a	25.17 a	45.89 a	17.71 a	15.64 c
Variation source						
Cultivate (C)		ns	**	**	*	ns
SAP(S)		**	**	**	**	**
C × S		ns	*	*	ns	ns

Notes: Mean ± standard deviation. Values followed by different letters within the column are significantly different at *p* < 0.05. respectively. *, ** indicate significant differences at *p* < 0.05 and *p* < 0.01, respectively; ns indicates not significant. The same applies below. Treatment abbreviations: N0 (Flat planting + 0 kg ha^−1^ SAP); N1 (Flat planting + 60 kg ha^−1^ SAP); N2 (Flat planting + 90 kg ha^−1^ SAP); D0 (Ridge-furrow planting with furrow sowing + 0 kg ha^−1^ SAP); D1 (Ridge-furrow planting with furrow sowing + 60 kg ha^−1^ SAP); D2 (Ridge-furrow planting with furrow sowing + 90 kg ha^−1^ SAP).

**Table 2:** Analysis of variance on the effects of different treatments on the storage performance and yield of quinoa in 2024 and 2025.

	2024			2025		
	Y	TKW	GWPP	Y	TKW	GWPP
C	**	**	**	*	**	*
S	**	**	**	**	**	**
C × S	ns	**	ns	ns	ns	ns

Notes: *, ** indicate significant differences at *p* < 0.05 and *p* < 0.01, respectively; ns indicates not significant. C: Planting pattern; S: SAP application rate; C × S: Interaction between C and S. GY, grain yield; TKW, thousand-kernel weight; GWPP, kernel weight of single ear.

**Table 3:** Effects of different planting patterns and superabsorbent polymer (SAP) application rates on the quinoa population-level sink-source ratio in 2024 and 2025.

Years	Treatments	Grain Weight/ LAI (g/cm^2^)	Grain Number/ LAI (Grain/cm^2^)	Grain Weight/ Leaf Dry Weight (g/g)	Grain Number/ Leaf Dry Weight (Grain/g)
2024	N0	208.50	62,961.63	20.54	852.34
	N1	91.78	50,583.15	20.33	755.20
	N2	86.53	42,192.96	20.33	717.80
	D0	171.13	59,803.14	19.70	801.06
	D1	92.59	48,782.64	19.73	693.98
	D2	85.72	40,471.30	20.12	675.72
Variation source					
Cultivate (C)		*	ns	ns	ns
SAP(S)		**	**	ns	**
C × S		**	ns	ns	ns
2025	N0	135.84	63,764.80	15.44	644.31
	N1	113.19	48,107.05	15.50	585.36
	N2	112.54	45,994.23	16.08	584.33
	D0	123.00	56,003.16	15.02	607.96
	D1	113.50	47,169.01	15.91	587.59
	D2	102.14	40,221.70	16.19	566.87
Variation source					
Cultivate (C)		**	**	ns	ns
SAP(S)		**	**	*	**
C × S		ns	*	ns	ns

Notes: Mean ± standard deviation. * Indicates *p* < 0.05, and ** indicates *p* < 0.01 and ns indicates no statistical significance. The same applies below. Treatment abbreviations: N0 (Flat planting + 0 kg ha^−1^ SAP); N1 (Flat planting + 60 kg ha^−1^ SAP); N2 (Flat planting + 90 kg ha^−1^ SAP); D0 (Ridge-furrow planting with furrow sowing + 0 kg ha^−1^ SAP); D1 (Ridge-furrow planting with furrow sowing + 60 kg ha^−1^ SAP); D2 (Ridge-furrow planting with furrow sowing + 90 kg ha^−1^ SAP).

## Data Availability

The original contributions presented in this study are included in the article. Further inquiries can be directed to the corresponding author.
